# Author Correction: Earthquake energy dissipation in a fracture mechanics framework

**DOI:** 10.1038/s41467-024-49551-z

**Published:** 2024-06-17

**Authors:** David S. Kammer, Gregory C. McLaskey, Rachel E. Abercrombie, Jean-Paul Ampuero, Camilla Cattania, Massimo Cocco, Luca Dal Zilio, Georg Dresen, Alice-Agnes Gabriel, Chun-Yu Ke, Chris Marone, Paul Antony Selvadurai, Elisa Tinti

**Affiliations:** 1 Institute for Building Materials, ETH Zurich, Zurich, Switzerland; 2 School of Civil and Environmental Engineering, Cornell University, Ithaca, NY USA; 3 Boston University, Boston, MA USA; 4 Université Côte d’Azur, Observatoire de la Côte d’Azur, IRD, CNRS, Géoazur, Valbonne, France; 5 Department of Earth, Atmospheric, and Planetary Sciences, Massachusetts Institute of Technology, Cambridge, MA USA; 6 Istituto Nazionale di Geofisica e Vulcanologia, Rome, Italy; 7 Earth Observatory of Singapore, Nanyang Technological University, Singapore, Singapore; 8 Asian School of the Environment, Nanyang Technological University, Singapore, Singapore; 9 Helmholtz Centre Potsdam, GFZ German Research Centre for Geosciences, Potsdam, Germany; 10 Scripps Institution of Oceanography, UCSD, La Jolla, USA; 11 Ludwig-Maximilians-Universität München, Munich, Germany; 12 Department of Geosciences, The Pennsylvania State University, University Park, PA 16802 USA; 13 La Sapienza Universitá di Roma, P.le Aldo Moro 5, 00185 Roma, Italia; 14 Swiss Seismological Service, ETH Zurich, Zurich, Switzerland

**Keywords:** Seismology, Geophysics

Correction to: *Nature Communications* 10.1038/s41467-024-47970-6, published online 3 June 2024

The original version of this Article contained an error in the Acknowledgement Section, which incorrectly omitted the following: ‘Massimo Cocco participated in this work as Principal Investigator of the European Research Council (ERC) project FEAR (grant agreement No 856559) under the European Community’s Horizon 2020 Framework Programme. Paul Selvadurai, Elisa Tinti and Luca Dal Zilio participated in this work in the framework of the European Research Council (ERC) project FEAR (grant agreement No 856559) under the European Community’s Horizon 2020 Framework Programme.’ This has been corrected in both the PDF and HTML versions of the Article.

